# High ovarian GDF-8 levels contribute to elevated estradiol production in ovarian hyperstimulation syndrome by stimulating aromatase expression

**DOI:** 10.7150/ijbs.60332

**Published:** 2021-06-11

**Authors:** Lanlan Fang, Yang Yan, Sijia Wang, Yanjie Guo, Yiran Li, Qiongqiong Jia, Xiaoyu Han, Boqun Liu, Jung-Chien Cheng, Ying-Pu Sun

**Affiliations:** 1 Center for Reproductive Medicine, Henan Key Laboratory of Reproduction and Genetics, The First Affiliated Hospital of Zhengzhou University, Zhengzhou, China.; 2 Department of Obstetrics & Gynaecology, The Chinese University of Hong Kong, New Territories, Hong Kong, China.

**Keywords:** GDF-8, Aromatase, Estradiol, OHSS, Granulosa cells

## Abstract

**Rationale:** Growth differentiation factor-8 (GDF-8), also known as myostatin, belongs to the transforming growth factor-beta (TGF-β) superfamily. GDF-8 is expressed in the ovary and regulates various ovarian functions. Ovarian hyperstimulation syndrome (OHSS) is one of the most serious disorders during *in vitro* fertilization treatment. Aromatase, encoded by the *CYP19A1* gene, is the enzyme that catalyzes the final step in estradiol (E2) biosynthesis. It has been demonstrated that high serum E2 levels are associated with the development of OHSS. However, the effects of GDF-8 on aromatase expression and its roles in the pathogenesis of OHSS remain unclear.

**Methods:** The effect of GDF-8 on aromatase expression and the underlying mechanisms were explored by a series of *in vitro* experiments in primary human granulosa-lutein (hGL) and KGN cells. Rat OHSS model and human follicular fluid samples were used to examine the roles of the GDF-8 system in the pathogenesis of OHSS.

**Results:** We demonstrate that GDF-8 stimulates aromatase expression and E2 production in hGL and KGN cells. In addition, TGF-β type I receptor ALK5-mediated SMAD2/3 signaling is required for GDF-8-induced aromatase expression and E2 production. Using a rat OHSS model, we show that the aromatase and GDF-8 levels are upregulated in the ovaries of OHSS rats. Blocking the function of ALK5 by the administration of its inhibitor, SB431542, alleviates OHSS symptoms and the upregulation of aromatase. Clinical results reveal that the protein levels of GDF-8 are upregulated in the follicular fluid of OHSS patients. Moreover, the expression of GDF-8 is increased in hGL cells of OHSS patients.

**Conclusions:** This study helps to elucidate the mechanisms mediating the expression of aromatase in human granulosa cells, which may lead to the development of alternative therapeutic approaches for OHSS.

## Introduction

Growth differentiation factor-8 (GDF-8), also known as myostatin, was first identified in 1997 and was discovered as a member of the TGF-β superfamily. GDF-8 gene knockout mice exhibit a dramatic increase in muscle mass, suggesting its negative regulatory role in skeletal muscle growth 1. Naturally occurring gene mutations or gene knockout models further confirm the inhibitory effect of GDF-8 on myogenesis in several species, including humans 2-5. GDF-8 is a secreted protein that is mainly synthesized by skeletal muscle cells. After secretion, the activity of circulating GDF-8 can be either increased or decreased by different factors 6. Although it remains controversial, GDF-8 has been shown to regulate adipogenesis outside the skeletomuscular system, and aberrant expression of GDF-8 is associated with obesity 7.

Increasing evidence has suggested the roles of GDF-8 in female reproductive functions as the expression of GDF-8 and its receptors have been detected in the ovary, uterus, and placenta 8-10. In the ovary, the ovarian follicle is the basic functional unit, which consists of an oocyte surrounded by granulosa and theca cells. Granulosa cells are essential for normal oocyte development and steroid hormone production. We and other groups have shown that GDF-8 and its receptors are expressed in human granulosa cells. In addition, secreted GDF-8 has been detected in human ovarian follicular fluid 10-13. Functionally, we have demonstrated that granulosa cell proliferation, steroidogenesis, cumulus expansion, and oocyte maturation are regulated by GDF-8 11-17. Collectively, these studies demonstrate that GDF-8 acts as an important local factor and can regulate various ovarian functions in an autocrine and/or paracrine fashion.

Controlled ovarian hyperstimulation (COH) is an approach that is generally applied to infertile women to produce more oocytes during assisted reproductive technology treatments. Ovarian hyperstimulation syndrome (OHSS), one of the serious complications associated with COH, is mainly caused by the administration of exogenous gonadotropins for ovarian stimulation and subsequent ovulation induction by the human chorionic gonadotropin (hCG) 18. Spontaneous OHSS rarely occurs in women who are not undergoing ovulation induction therapies. Therefore, OHSS is considered an iatrogenic complication. However, severe OHSS may lead to maternal death 19-21. To date, several risk factors have been reported to be associated with the development of OHSS. Among them, high serum estradiol (E2) levels before administration of hCG are significantly associated with the development of OHSS. In addition, inhibition of E2 levels prevents OHSS development 22-24.

E2 is the most active form of natural estrogens and is known to play important roles in female reproduction 25. It is well characterized that the aromatase enzyme, which is encoded by the cytochrome P450 family 19 subfamily A member 1 (*CYP19A1*) gene, plays a pivotal role in the biosynthesis of E2. In the ovarian follicle, aromatase is expressed in granulosa cells, not in theca cells. According to the two-cell-two-gonadotropin theory, by converting theca cell-derived testosterone via aromatase, E2 is synthesized and produced by ovarian granulosa cells 26. After ovulation, granulosa cells differentiate into granulosa-lutein cells. The expression of aromatase is detected in human granulosa-lutein (hGL) cells and contributes to E2 production in the early stage of pregnancy and the luteal phase of the menstrual cycle 27, 28. Although the underlying molecular mechanisms remain undefined, we have shown that GDF-8 treatment increases aromatase expression in hGL cells 14. However, whether GDF-8 levels vary between normal and OHSS patients remains unknown. In the present study, we investigated the underlying molecular mechanisms of GDF-8 involved in aromatase expression in hGL cells. We also examined the expression of GDF-8 in the OHSS rat model and OHSS patients.

## Materials and Methods

### Antibodies and reagents

The aromatase antibody was purchased from Bio-Rad Laboratories (#MCA2077). The phospho-SMAD2 (#3108), phospho-SMAD3 (#9520), SMAD2 (#3103), SMAD3 (#9523), and SMAD4 (#38454) antibodies were purchased from Cell Signaling Technology. The α-tubulin antibody (#sc-23948) was purchased from Santa Cruz Biotechnology. The recombinant human GDF-8 was obtained from R&D systems. The SB431542 was obtained from Sigma.

### Human follicular fluid samples

The present study received approval and was performed in accordance with the approved guidelines from the Zhengzhou University Research Ethics Board. Written informed consent was obtained from all patients before collecting clinical samples. None of the women had been prescribed any medications before enrollment. Human follicular fluid samples were obtained from 50 women (25 control and 25 OHSS patients) during *in vitro* fertilization treatment. The causes of infertility were tubal obstruction or male infertility. Patients with polycystic ovary syndrome, endometriosis, diminished ovarian reserve, chromosome abnormality, or hydrosalpinx were excluded from this study. All patients were treated with a standard long protocol. At the mid-luteal phase, the gonadotropin-releasing hormone agonist, triptorelin (0.1 mg; Ipsen Pharma Biotech), was subcutaneously administered daily. Approximately 14 days after the injection of the GnRH agonist, recombinant FSH (Gonal-F; Merck) at a dosage of 150-300 IU was administered daily. When at least three follicles had reached 18 mm, hCG (10000 IU, Livzon) was injected. Oocyte retrieval was scheduled approximately 34-36 h after hCG injection by transvaginal ultrasound-guided follicular aspiration. Follicular fluid was collected when the oocytes were retrieved. Only the first follicular fluid aspirate without blood or flushing solution was used for analysis. After 10 min of centrifugation at 2000 rpm, the supernatant was stored at -80 °C until further analysis.

### Cell culture

Primary human granulosa-lutein (hGL) cells were purified by density centrifugation from follicular aspirates collected from women undergoing oocyte retrieval as previously described 29. The human granulosa cell tumor-derived cell line, KGN 30, was kindly provided by Dr. Aaron Hsueh at Stanford University. Cells were cultured in a humidified atmosphere containing 5% CO2 and 95% air at 37 °C in phenol-red free Dulbecco's modified Eagle's medium/nutrient mixture F-12 Ham medium (DMEM/F-12; Gibco) supplemented with 10% charcoal/dextran-treated FBS (HyClone), 100 U/mL penicillin and 100 μg/mL streptomycin sulfate (Boster). Primary hGL cells were cultured in 12-well plates at a density of 10^5^ cells/cm^2^ with 1 mL of culture medium for 5 days. After 5 days of culture, primary hGL cells were serum-starved in a medium containing 0.5% charcoal/dextran-treated FBS for 24 h to induce quiescence before treatments. All treatments for primary hGL cells were performed in a medium containing 0.5% charcoal/dextran-treated FBS. KGN cells were cultured in 6-well plates with 2 mL of culture medium. KGN cells were grown to 80% confluence and serum-starved in a medium without FBS for 24 h to induce quiescence before treatments. All treatments for KGN cells were performed in a medium without FBS.

### Rat OHSS model

Female Wistar rats were obtained from Charles River Laboratories (Beijing, China). Animal handling was in accordance with the Guide for the Care and Use of Laboratory Animals published by the US National Institutes of Health. The rats were housed in an environmentally controlled room with free access to food and water. Animal studies were approved by the Zhengzhou University Animal Research Ethics Board. The rat OHSS model was established according to a previous study 31. PMSG (50 IU/d) was administered i.p. for 4 consecutive days to 4-week-old Wistar female rats followed by hCG administration (25 IU, i.p.) on the fourth day. Control rats were administered a single dose of PMSG (10 IU) followed by hCG (10 IU) 48 h later. Rats were treated with vehicle control (DMSO) or SB431542 (10 mg/kg, i.p.) on days 4-6. All rats were euthanized on day 7. Each group contained 5 rats. Changes in body weight and ovarian weight were recorded.

### Reverse transcription quantitative real-time PCR (RT-qPCR)

Total RNA was extracted with the RNeasy Plus Mini Kit (QIAGEN) according to the manufacturer's instructions. RNA (1 μg) was reverse-transcribed into first-strand cDNA with the iScript Reverse Transcription Kit (Bio-Rad Laboratories). Each 20 μL qPCR reaction contained 1X SYBR Green PCR Master Mix (Applied Biosystems), 60 ng of cDNA and 250 nM of each specific primer. The following primers were used: human *CYP19A1* aromatase, 5'-GAG AAT TCA TGC GAG TCT GGA-3' (sense) and 5'-CAT TAT GTG GAA CAT ACT TGA GGA CT-3' (antisense); human *ALK4*, 5'-TCT CTC CAC CTC AGG GTC TG-3' (sense) and 5'-GCC ATA CTT CCC CAA ACC GA-3' (antisense); human *ALK5*, 5'-GTT AAG GCC AAA TAT CCC AAA CA-3' (sense) and 5'-ATA ATT TTA GCC ATT ACT CTC AAG G-3' (antisense); human *SMAD4*, 5'-TCC ACA GGA CAG AAG CCA TT-3' (sense) and 5'-GTC ACT AAG GCA CCT GAC CC-3' (antisense); human *GAPDH*, 5'-GAG TCA ACG GAT TTG GTC GT-3' (sense) and 5'-GAC AAG CTT CCC GTT CTC AG-3' (antisense); rat *CYP19A1* aromatase, 5'-GCT GGA CAC TTC TAA CAC GC-3' (sense) and 5'-ATA AGG AGT GCT TGC CAG GC-3' (antisense); rat *GDF-8*, 5'-TAA CCT TCC CAG GAC CAG GA-3' (sense) and 5'-CAC TCT CCA GAG CAG TAA TT-3' (antisense); and rat *GAPDH*, 5'-GAC ATG CCG CCT GGA GAA AC-3' (sense) and 5'-AGC CCA GGA TGC CCT TTA GT-3' (antisense). RT-qPCR was performed using an Applied Biosystems QuantStudio 12K Flex Real-Time PCR system equipped with a 96-well optical reaction plate. The specificity of each assay was validated by melting curve analysis and agarose gel electrophoresis of the PCR products. All of the RT-qPCR experiments were run in triplicate, and a mean value was used to determine the mRNA levels. Water and mRNA without reverse transcriptase (RT) were used as negative controls. Relative quantification of the mRNA levels was performed using the comparative Ct method with GAPDH as the reference gene and using the 2^-∆∆Ct^ formula.

### Western blot analysis

Cells were lysed in cell lysis buffer (Cell Signaling Technology) supplemented with a protease inhibitor cocktail (Sigma). Equal amounts (50 µg) of protein were separated by SDS polyacrylamide gel electrophoresis and transferred onto PVDF membranes. After 1 h of blocking with 5% nonfat dry milk in Tris-buffered saline (TBS), the membranes were incubated overnight at 4 °C with primary antibodies diluted in 5% nonfat milk/TBS. The dilutions for antibodies were: aromatase (500x); p-SMAD2 (1000x); p-SMAD3 (1000x); SMAD2 (1000x); SMAD3 (1000x); SMAD4 (1000x), and α-Tubulin (5000x). Following primary antibody incubation, the membranes were incubated with appropriate HRP-conjugated secondary antibodies. Immunoreactive bands were detected using an enhanced chemiluminescent substrate (Bio-Rad Laboratories) and imaged with a ChemiDoc MP Imager (Bio-Rad Laboratories).

### Small interfering RNA (siRNA) transfection

To knockdown endogenous *ALK4*, *ALK5* or *SMAD4*, cells were transfected with 50 nM ON-TARGETplus SMARTpool siRNA targeting a specific gene (Dharmacon) using Lipofectamine RNAiMAX (Invitrogen). The siCONTROL NON-TARGETING pool siRNA (Dharmacon), was used as the transfection control.

### Measurement of amphiregulin, GDF-8, and estradiol

Amphiregulin and GDF-8 levels in human follicular fluid were measured using an enzyme-linked immunosorbent assay (ELISA). Amphiregulin and GDF-8 ELISA kits (R&D Systems) were used in accordance with the manufacturer's protocol. Estradiol (E2) levels in culture media were also measured by ELISA. A human E2 ELISA kit (Cayman) was used in accordance with the manufacturer's protocol. E2 levels in the culture media were normalized to the protein concentrations from the cell lysates. Normalized E2 values in the culture media from the treatments are represented as relative values by comparison to the control treatment.

### Statistical analysis

The results are presented as the mean ± SEM of at least three independent experiments. The animal and clinical results are presented as the mean ± SD. All statistical analyses were analyzed by PRISM software. Multiple comparisons were analyzed using one-way ANOVA followed by Tukey's multiple comparison test. For experiments involving only two groups, the results were analyzed by *t* test. A significant difference was defined as *p*<0.05.

## Results

### The expression of aromatase is upregulated by GDF-8 in hGL cells

**Figure 1:**
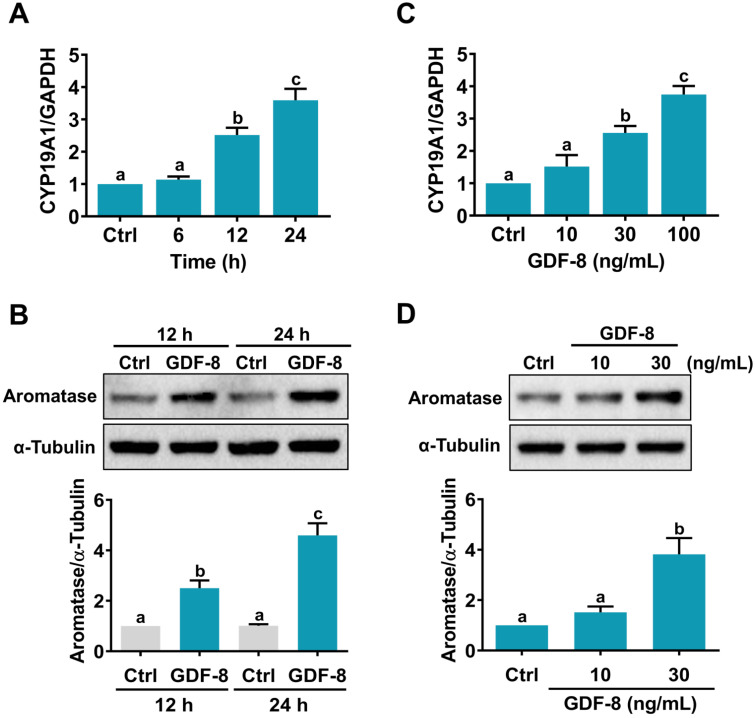
**GDF-8 stimulates aromatase expression in hGL cells.** A and B, Cells were treated with 100 ng/mL GDF-8 for different periods, and the mRNA (A) and protein (B) levels of aromatase (*CYP19A1*) were examined by RT-qPCR and western blot, respectively. The level of aromatase mRNA at each time point was normalized to the GAPDH mRNA level at the same time point. C and D, Cells were treated with 10, 30, or 100 ng/mL GDF-8 for 24 h, and the mRNA (C) and protein (D) levels of aromatase (*CYP19A1*) were examined by RT-qPCR and western blot, respectively. The results are expressed as the mean ± SEM of at least three independent experiments. The values without a common letter are significantly different (*p*<0.05).

We have previously shown that GDF-8 enhances follicle-stimulating hormone (FSH)-stimulated aromatase expression in primary cultures of hGL cells 14. However, it remains unknown how GDF-8 directly stimulates aromatase expression in hGL cells. Consistent with our previous results, the mRNA levels of aromatase (*CYP19A1*) were upregulated by the treatment of 100 ng/mL human recombinant GDF-8 in a time-dependent manner (Figure 1A). Western blot results confirmed the stimulatory effect of GDF-8 on aromatase protein levels (Figure 1B). We also examined the effect of different concentrations of GDF-8 on aromatase expression. As shown in Figure 1C, treatment with 10 ng/mL GDF-8 did not affect the mRNA levels of aromatase. A significant stimulatory effect was obtained after treating cells with 30 or 100 ng/mL GDF-8. The stimulatory effect of 30 ng/mL GDF-8 on aromatase protein levels was further confirmed by western blot analysis (Figure 1D). Therefore, 30 ng/mL GDF-8 was applied in the subsequent experiments.

### ALK5 is required for GDF-8-induced aromatase expression

**Figure 2:**
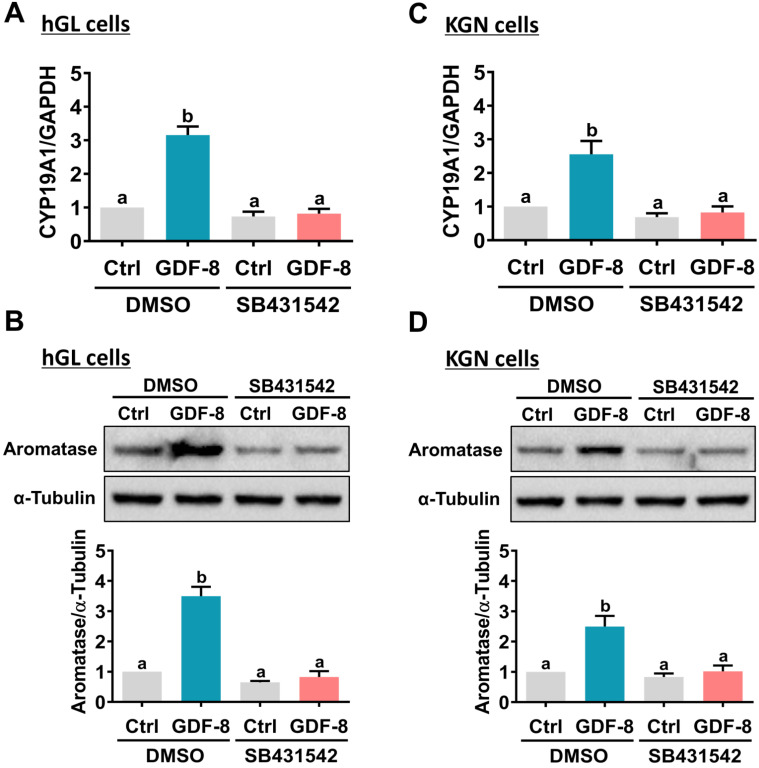
**Pharmacological inhibition of ALK4/5 blocks GDF-8-induced aromatase expression in hGL and KGN cells.** A and B, hGL cells were pretreated with vehicle control (DMSO) or 10 µM SB431542 for 1 h, and then treated with 30 ng/mL GDF-8 for 24 h. Aromatase mRNA levels (*CYP19A1*) (A) and protein levels (B) were examined by RT-qPCR and western blot, respectively. C and D, KGN cells were pretreated with vehicle control (DMSO) or 10 µM SB431542 for 1 h, and then they were treated with 30 ng/mL GDF-8 for 24 h. The aromatase mRNA levels (*CYP19A1*) (C) and protein levels (D) were examined by RT-qPCR and western blot, respectively. The results are expressed as the mean ± SEM of at least three independent experiments. The values without a common letter are significantly different (*p*<0.05).

**Figure 3:**
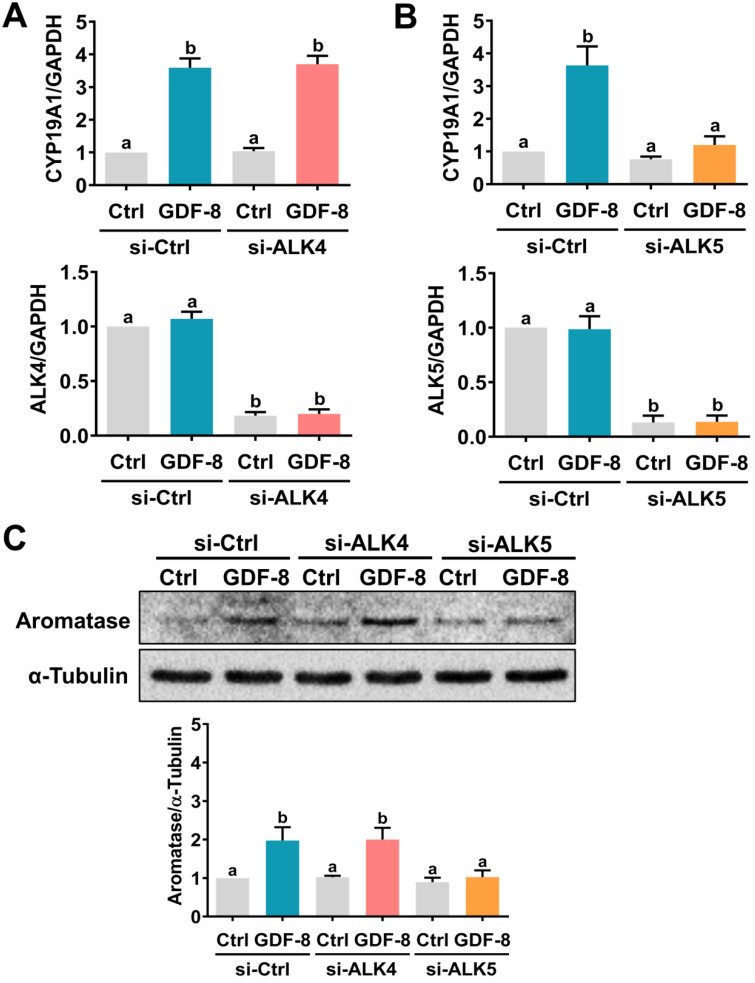
** ALK5 but not ALK4 is involved in GDF-8-induced aromatase expression.** A, KGN cells were transfected with 50 nM control siRNA (si-Ctrl) or ALK4 siRNA (si-ALK4) for 48 h, and then treated with 30 ng/mL GDF-8 for 24 h. The mRNA levels of aromatase (*CYP19A1*) and ALK4 were examined by RT-qPCR. B, KGN cells were transfected with 50 nM control siRNA (si-Ctrl) or ALK5 siRNA (si-ALK5) for 48 h, and then treated with 30 ng/mL GDF-8 for 24 h. The mRNA levels of aromatase (*CYP19A1*) and ALK5 were examined by RT-qPCR. C, KGN cells were transfected with 50 nM control siRNA (si-Ctrl), ALK4 siRNA (si-ALK4), or ALK5 siRNA (si-ALK5) for 48 h, and then treated with 30 ng/mL GDF-8 for 24 h. The protein levels of aromatase were examined by western blot. The results are expressed as the mean ± SEM of at least three independent experiments. The values without a common letter are significantly different (*p*<0.05).

It has been implicated that the TGF-β type I receptors, ALK4 and ALK5, are putative receptors for GDF-8 that mediate its biological functions 32. To examine the involvement of ALK4 and ALK5 in GDF-8-stimulated aromatase expression, SB431542, a potent ALK4/5/7 inhibitor, was used to block the function of ALK4 and ALK5 33. As shown in Figures 2A and 2B, the stimulatory effects of GDF-8 on aromatase mRNA and protein levels were blocked by pretreatment with SB431542. We also examined the effect of GDF-8 on KGN cells. The KGN cell line is derived from human ovarian granulosa cell tumors, but KGN cells preserve various physiological functions of normal granulosa cells, including the expression of functional FSH receptor and the expression and activity of aromatase 30. To date, the KGN cell line has been widely used as a cell model for understanding the regulation of aromatase expression and E2 production. Similar to the results obtained from hGL cells, treatment with GDF-8 stimulated both the mRNA and protein levels of aromatase in KGN cells. These stimulatory effects were blocked by pretreatment with SB431542 (Figures 2C and 2D). Because SB431542 blocks both ALK4 and ALK5, ALK4 and ALK5 siRNAs were used to knockdown the expression of a specific gene to further explore the involvement of ALK4 and ALK5 in GDF-8-stimulated aromatase expression. To make the experiments more technically feasible, particularly those involving gene knockdowns, KGN cells were used as the experimental model. Transfection of KGN cells with ALK4 siRNA downregulated endogenous mRNA levels of ALK4. However, knockdown of ALK4 did not affect the stimulatory effect of GDF-8 on aromatase mRNA levels (Figure 3A). Interestingly, the stimulatory effect of GDF-8 on aromatase mRNA levels was blocked by the knockdown of ALK5 (Figure 3B). The same results of the involvement of ALK5, but not ALK4, in GDF-8-stimulated aromatase protein levels were observed by western blot analysis (Figure 3C). Collectively, these results indicate that the stimulatory effect of GDF-8 on aromatase expression in human granulosa cells is mediated by ALK5.

### GDF-8 upregulates aromatase expression and induces E2 production by activating the ALK5-mediated SMAD2/3 signaling pathway

**Figure 4:**
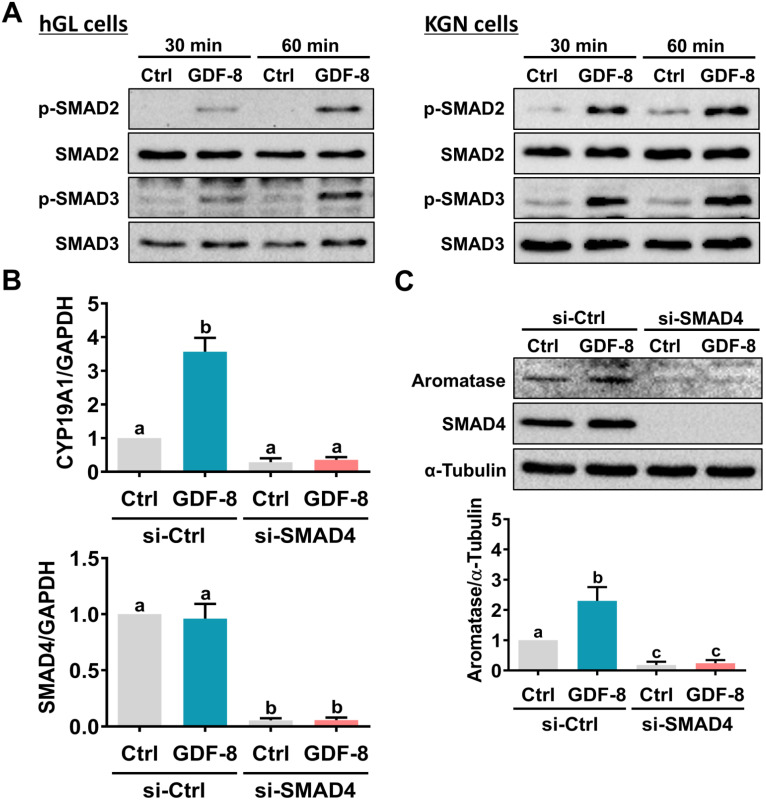
**SMAD2/3 signaling pathways are involved in GDF-8-induced aromatase expression.** A, hGL cells (left panel) and KGN cells (right panel) were treated with 30 ng/mL GDF-8 for 30 and 60 min. The levels of phosphorylated and total forms of SMAD2 and SMAD3 were determined by western blot. B and C, KGN cells were transfected with 50 nM control siRNA (si-Ctrl) or SMAD4 siRNA (si-SMAD4) for 48 h, and then treated with 30 ng/mL GDF-8 for 24 h. The mRNA (B) and protein (C) levels of aromatase (*CYP19A1*) and SMAD4 were examined by RT-qPCR and western blot, respectively. The results are expressed as the mean ± SEM of at least three independent experiments. The values without a common letter are significantly different (*p*<0.05).

**Figure 5:**
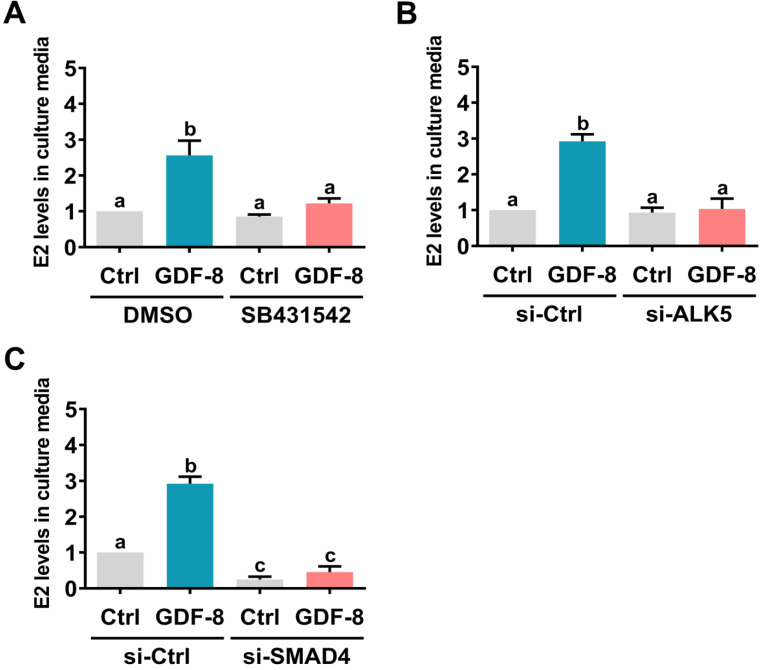
** ALK5-mediated SMAD signaling is involved in GDF-8-induced E2 production.** A, KGN cells were pretreated with vehicle control (DMSO) or 10 µM SB431542 for 1 h, and then they were treated with 30 ng/mL GDF-8 every 24 h for 48 h. B and C, KGN cells were transfected with 50 nM control siRNA (si-Ctrl), ALK5 siRNA (si-ALK5) (B), or SMAD4 siRNA (si-SMAD4) (C) for 48 h, and then treated with 30 ng/mL GDF-8 every 24 h for 48h. E2 levels in culture media were examined by ELISA. The results are expressed as the mean ± SEM of at least three independent experiments. The values without a common letter are significantly different (*p*<0.05).

SMAD2 and SMAD3 are well characterized downstream signaling pathways of ALK5 32. Western blot analysis showed that treatment with GDF-8 activated both the SMAD2 and SMAD3 signaling pathways in hGL and KGN cells (Figure 4A). To examine whether SMAD signaling is involved in GDF-8-induced aromatase expression, endogenous SMAD4 was knocked down by siRNA transfection because SMAD4 is necessary for SMAD-dependent signaling pathways 34. As shown in Figures 4B and 4C, transfection of SMAD4 siRNA significantly downregulated endogenous SMAD4 mRNA and protein levels. Importantly, knockdown of SMAD4 not only decreased basal aromatase mRNA and protein levels but also blocked the stimulatory effects of GDF-8 on aromatase mRNA and protein levels. Given the pivotal role of aromatase in E2 synthesis, we next examined whether GDF-8 affects E2 production in KGN cells. As shown in Figure 5A, treatment with GDF-8 induced E2 production, and this effect was abolished by pretreatment with SB431542. In addition, the stimulatory effect of GDF-8 on E2 production was blocked by the knockdown of ALK5 and SMAD4 (Figures 5B and 5C).

### Inhibition of ALK5 attenuates the pathogenesis of OHSS in rats

**Figure 6:**
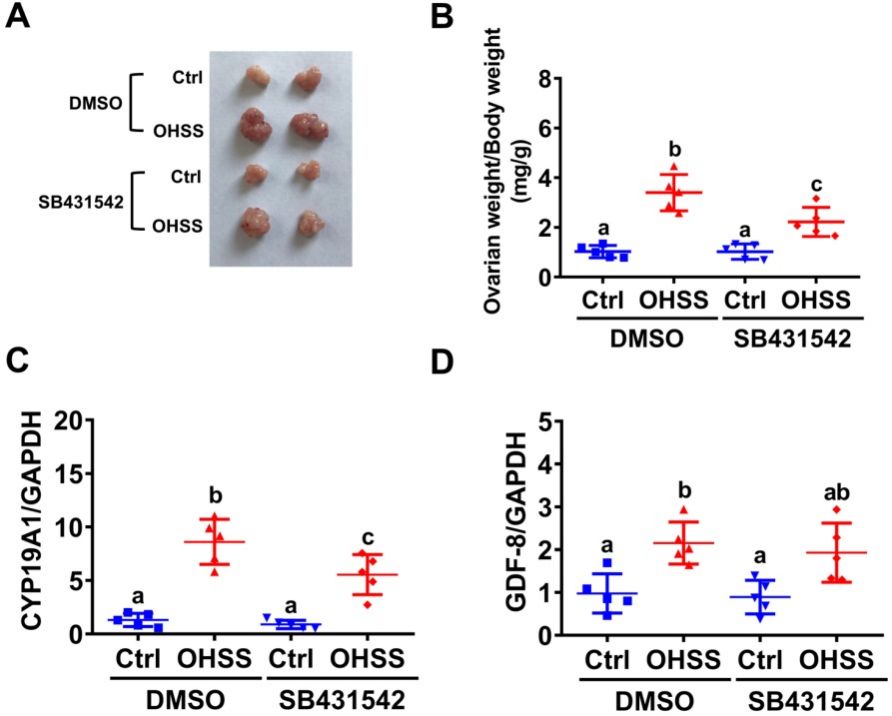
** Administration of SB431542 attenuates OHSS symptoms.** A, Representative ovaries were photographed from each group (n=5 for each group). B, Ovarian weight over body weight was determined after rats were euthanized. C and D, aromatase (*CYP19A1*) (C) and GDF-8 (D) mRNA levels in rat ovaries were examined by RT-qPCR. The results are expressed as the mean ± SD. Values without a common letter are significantly different (*p*<0.05).

To further examine the role of GDF-8 in the pathogenesis of OHSS, SB431542 was applied to block the function of GDF-8 in a rat OHSS model. Consistent with our previous study 31, induction of OHSS significantly enlarged the size of the ovary and increased ovarian weight in rats. Administration of SB431542 attenuated the increases in ovarian size and weight in the OHSS group (Figures 6A and 6B). RT-qPCR results showed that the mRNA levels of aromatase were significantly upregulated compared to the control group and this induction was attenuated by the administration of SB431542 (Figure 6C). Importantly, our results also showed that the mRNA levels of GDF-8 were upregulated in the ovaries of OHSS rats. However, the upregulation of GDF-8 mRNA levels was not affected by the administration of SB431542 (Figure 6D).

### GDF-8 levels are elevated in follicular fluid and granulosa cells of OHSS patients

**Figure 7:**
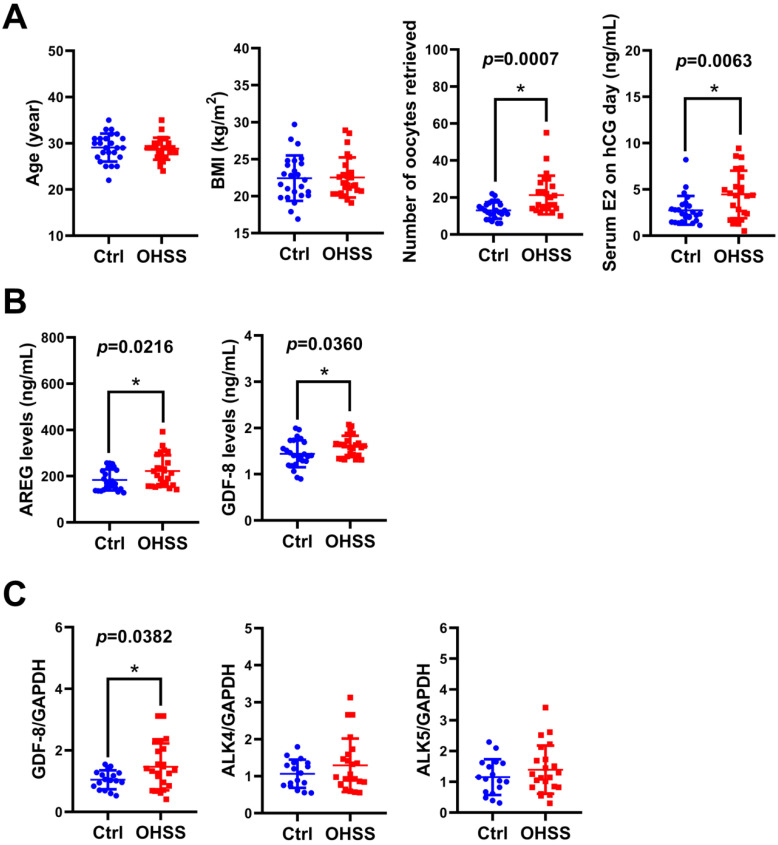
**GDF-8 levels are upregulated in the follicular fluid and hGL of OHSS patients.** A, The age, BMI, number of oocytes retrieved, and serum E2 levels on hCG day were summarized. B, AREG and GDF-8 protein levels in the follicular fluid of control (n=25) and OHSS (n=25) patients were examined by ELISA. C, The mRNA levels of GDF-8, ALK4, and ALK5 in hGL cells of control (n=17) and OHSS patients (n=22) were examined by RT-qPCR. The results are expressed as the mean ± SD. The values without a common letter are significantly different (*p*<0.05).

Given the critical role of E2 in the pathogenesis of OHSS, we examined the levels of GDF-8 in the follicular fluid of 25 control and 25 OHSS patients. As shown in Figure 7A, age and BMI did not vary significantly between control and OHSS patients. As expected, the number of oocytes retrieved and serum E2 levels on hCG administration day were significantly higher in OHSS patients than in control patients. We have previously shown that the levels of the EGFR ligand, amphiregulin (AREG), are increased in the follicular fluid of OHSS patients 35. Consistent with our previous results, AREG protein levels were higher in the follicular fluid of OHSS patients than in that of control patients. Interestingly, GDF-8 protein levels were upregulated in the follicular fluid of OHSS patients (Figure 7B). We also examined the expression of GDF-8, ALK4, and ALK5 in the hGL cells derived from control and OHSS patients. RT-qPCR results showed that the mRNA levels of GDF-8 were higher in hGL cells of OHSS patients than in those of control patients. Both ALK4 and ALK5 mRNA levels in hGL cells did not vary significantly between control and OHSS patients (Figure 7C).

## Discussion

Increasing evidence has indicated that the function of granulosa cells depends not only on endocrine regulators but also on a variety of locally produced factors that exert their effects in an autocrine and/or paracrine fashion. Steroid hormone production is one of the major biological functions of ovarian granulosa cells. Our previous studies have demonstrated that steroidogenic acute regulatory protein (StAR) is downregulated by GDF-8 treatment in hGL cells 17. However, other steroidogenesis-related enzymes such as P450 side chain cleavage enzyme (P450scc) and 3β-hydroxysteroid dehydrogenase (3β-HSD) are not affected by GDF-8 in hGL cells 13. Some hormones and growth factors expressed in the human ovary and follicular fluid have been shown to stimulate aromatase expression or E2 production in hGL cells 29, 36, 37. GDF-8 belongs to the TGF-β superfamily which plays important role in the regulation of ovarian function 38. To date, the expression of several TGF-β superfamily members has been detected in the ovary. However, only a few studies have examined the direct effect of TGF-β superfamily members on aromatase expression. In KGN cells, activin treatment stimulates aromatase expression 39. In human granulosa cells, aromatase expression is induced by BMP2 40. In bovine granulosa cells, GDF-8 increases basal aromatase expression and E2 production 41. In this study, we showed that treatment of primary hGL cells and KGN cells with GDF-8 stimulated aromatase expression. Collectively, our findings together with previous studies reveal the critical autocrine/paracrine roles of locally produced factors in the regulation of aromatase expression and E2 production in human granulosa cells. In addition, our study suggests that GDF-8-induced aromatase expression and E2 production could be potential therapeutic targets for the treatment of OHSS.

ALK4 and ALK5 have been reported to be type I TGF-β receptors for GDF-8 as GDF-8 inhibits adipogenesis through ALK4- and ALK5-mediated activation of SMAD2/3 signaling pathways 42. Our previous studies have shown that GDF-8 inhibits the expression of StAR and pentraxin 3 through ALK5 11, 17. In the present study, we used a siRNA-mediated approach to reveal that the stimulatory effect of GDF-8 on aromatase expression in human granulosa cells was mediated by ALK5 but not ALK4. These results agreed with a previous study showing that ALK4 mediates the function of GDF-8 in myogenic cells, while its function in non-myogenic cells is mainly mediated by ALK5 43. In addition, we showed that both SMAD2 and SMAD3 were activated upon GDF-8 treatment. Knockdown of SMAD4 blocked GDF-8-stimulated aromatase expression. These results demonstrated the important role of SMAD2/3 signaling pathways in mediating the function of GDF-8 in human granulosa cells. In a context-dependent manner, SMAD2 and SMAD3 can redundantly or differentially mediate TGF-β signaling 44. It has been shown that GDF-8-inhibited StAR expression in hGL cells is mediated by SMAD3 but not SMAD2 17. Whether the same is true for GDF-8-stimulated aromatase expression remains unclear and warrants further investigation.

The gene expression of GDF-8 can be regulated by both transcriptional machinery and posttranslational machinery, but most of the mechanisms are identified in the myogenic context of animal models 45. In the present study, we found that the protein levels of GDF-8 were upregulated in the ovaries of the rat OHSS model and the follicular fluid of OHSS patients. In addition, hGL cells derived from OHSS patients expressed higher GDF-8 compared to those derived from controls. However, the mechanism that contributes to the elevation of GDF-8 expression in OHSS patients remains unknown. Similar to our previous study 46, the administration of SB431542 attenuated OHSS symptoms. However, SB431542 did not affect the increases of GDF-8 levels in the ovaries of OHSS rats. In ovariectomized rats, E2 has been shown to increase GDF-8 expression in the soleus muscle 47. Interestingly, E2 treatment does not affect GDF-8 mRNA levels in the muscle of postmenopausal women 48. To date, whether E2 regulates GDF-8 expression in nonmuscle cells remains unknown. Given the high levels of E2 in OHSS patients, it is possible that E2 may stimulate GDF-8 expression and contribute to the high GDF-8 levels observed in OHSS patients. Future studies are required to test this hypothesis. Single nucleotide polymorphisms (SNPs) have been identified in the promoter region of the GDF-8 gene in several animal species. SNPs affect the expression of GDF-8 by disrupting the binding of transcription factors 45. Mutations and SNPs of the human GDF-8 gene have been reported 3, 49. However, whether these factors contribute to the aberrant expression of GDF-8 in OHSS patients is unclear. Further work is needed to examine the signature of the GDF-8 gene in OHSS patients.

In summary, the present study reveals the stimulatory effect of GDF-8 on aromatase expression and E2 production in human granulosa cells. These effects are mediated by ALK5 and its downstream SMAD2/3 signaling. In addition, the expression of GDF-8 is upregulated in the follicular fluid and granulosa cells of OHSS patients, which leads to high aromatase expression and E2 levels and both of that subsequently contribute to the pathogenesis of OHSS. These results provide a better understanding of the mechanisms mediating the expression of aromatase and E2 production in human granulosa cells, which may lead to the development of alternative therapeutic approaches for OHSS.

## Abbreviations

3β-HSD: 3β-hydroxysteroid dehydrogenase
AREG: amphiregulin
COH: controlled ovarian hyperstimulation
ELISA: enzyme-linked immunosorbent assay
E2: estradiol
FSH: follicle-stimulating hormone
GDF-8: growth differentiation factor-8
hCG: human chorionic gonadotropin
hGL cells: human granulosa-lutein cells
IVF: *in vitro* fertilization
OHSS: ovarian hyperstimulation syndrome
P450scc: P450 side chain cleavage enzyme
SNPs: single nucleotide polymorphisms
siRNA: small interfering RNA
StAR: steroidogenic acute regulatory protein
TGF-β: transforming growth factor-beta
